# Compromised Hippocampal Neuroplasticity in the Interferon-α and Toll-like Receptor-3 Activation-Induced Mouse Depression Model

**DOI:** 10.1007/s12035-020-01927-0

**Published:** 2020-06-05

**Authors:** Eduardo H. Sanchez-Mendoza, Santiago Camblor-Perujo, Luiza Martins Nascentes-Melo, Egor Dzyubenko, Michael Fleischer, Tayana Silva de Carvalho, Linda-Isabell Schmitt, Markus Leo, Tim Hagenacker, Arne Herring, Kathy Keyvani, Sujoy Bera, Natalia Kononenko, Christoph Kleinschnitz, Dirk M. Hermann

**Affiliations:** 1 Department of Neurology, University Hospital Essen, Hufelandstr. 55, D-45122 Essen, Germany; 2 Department of Pathology and Neuropathology, University Hospital Essen, Hufelandstr. 55, D-45122 Essen, Germany; 3 Cologne Excellence Cluster for Cellular Stress Responses in Aging-Associated Diseases (CECAD), University of Cologne, Hufelandstr. 55, D-45122 Essen, Germany

**Keywords:** Dendritic plasticity, Major depressive disorder, Neuronal depolarization, Neuronal plasticity, Synaptic plasticity, Patch clamp recording

## Abstract

Disrupted neuronal plasticity due to subtle inflammation is considered to play a fundamental role in the pathogenesis of major depressive disorder. Interferon-α (IFN-α) potentiates immune responses against viral pathogens that induce toll-like receptor-3 (TLR3) activation but evokes severe major depressive disorder in humans by mechanisms that remain insufficiently described. By using a previously established mouse model of depression induced by combined delivery of IFN-α and polyinosinic:polycytidylic acid (poly(I:C)), a TLR3 agonist, we provide evidence that IFN-α and poly(I:C) reduce apical dendritic spine density in the hippocampal CA1 area ex vivo via mechanisms involving decreased TrkB signaling. In vitro, IFN-α and poly(I:C) treatments required neuronal activity to reduce dendritic spine density and TrkB signaling. The levels of presynaptic protein vesicular glutamate transporter (VGLUT)-1 and postsynaptic protein postsynaptic density-95 (PSD95) were specifically decreased, whereas the expression of both synaptic and extrasynaptic α-amino-3-hydroxy-5-methyl-4-isoxazolepropionic acid receptor 1 (AMPAR1) was increased by IFN-α and poly(I:C) delivery. Patch clamp recordings in primary hippocampal neurons revealed that morphological changes at the synapse induced by IFN-α and poly(I:C) costimulation were accompanied by an increased action potential threshold and action potential frequency, indicative of impaired neuronal excitability. Taken together, IFN-α and poly(I:C) delivery leads to structural and functional alterations at the synapse indicating that compromised neuroplasticity may play an integral role in the pathogenesis of immune response-induced depression.

**Electronic supplementary material:**

The online version of this article (10.1007/s12035-020-01927-0) contains supplementary material, which is available to authorized users.

## Introduction

Approximately 30 to 50% of patients suffering from chronic hepatitis C virus (HCV) who are treated with IFN-α develop severe depression along with confusion states, paranoia, and suicidal ideation [1, 2], which is in many cases a cause of therapy discontinuation [1, 3]. Nevertheless, the mechanisms of how the combined action of IFN-α and double-stranded RNA viruses may induce mood disorders remain poorly understood. Apart from the inflammatory response induced by any infectious viral organism, double-stranded RNA viruses induce pronounced TLR3 activation. We previously showed that IFN-α and TLR3 activation by poly(I:C) regulates the neuronal expression of a broad set of depression-associated genes in the hippocampus and prefrontal cortex of mice that were also found to be regulated in IFN-α-treated depressed patients and which correlated with depressive behavior in mice [1, 4]. Not surprisingly, we also showed that combined delivery of IFN-α and poly(I:C) induced localized inflammatory responses within the prefrontal cortex and hippocampus [4].

The regulation of growth, maturation, or pruning of dendritic spines and synaptic contacts and functional organization of neuronal networks is essential for the establishment of physiological behaviors [3, 5]. In fact, reduced or increased density of dendritic spines is associated with depressive behavior in mice lacking efficient synaptic pruning [6]. The glutamatergic system regulates neuronal sprouting, dendritic pruning, and synaptic contact formation through the regulation of the expression of brain-derived neurotrophic factor (BDNF) which in turn activates subcellular signaling pathways through its receptor tropomyosin receptor kinase B (TrkB) [7, 8]. Thus, compromised neuronal plasticity and communication in response to neurotransmitter release may contribute to depression pathogenesis.

TLRs, including TLR3, and other components of the innate immune system are now being recognized to play a role in neuronal plasticity [9], neuronal growth, and memory formation [1, 10–12]. IFN-α potentiates immune responses to double-stranded viruses by activation of the Janus kinase/signal transduction and activator of transcription-1 (STAT1) pathway, which amplifies IFN-α expression and the expression of other genes involved in growth arrest, cell death, and positive and negative regulators of antiviral responses [3].

Based on these insights, we asked whether IFN-α and poly(I:C)-induced depression is caused by compromised hippocampal plasticity due to alterations to the glutamatergic system. Using ex vivo studies in the IFN-α and poly(I:C)-induced depression paradigm [4], which we combined with in vitro studies in primary hippocampal neurons, we characterized structural and functional correlates of neuronal plasticity changes.

## Materials and Methods

### Animals

C57BL6/j mice (Harlan, Indianapolis, IN, USA) were used throughout the study. In ex vivo experiments, male 8–12-week-old mice were used. For in vitro studies, primary hippocampal cell cultures were obtained from P1–5 pups (morphological studies following enhanced green fluorescent protein [EGFP] transfection) or E15 embryos (all other studies). Experiments were performed in accordance with the National Institutes of Health Guidelines for the Care and Use of Laboratory Animals with local government approval (Bezirksregierung Düsseldorf, TSG966/08 and TSG1490/15).

### Intraventricular IFN-α and Poly(I:C) Delivery

Mice were anesthetized with 1% isoflurane (30% O_2_, remainder N_2_O) and placed in a stereotactic frame. The skin overlying the skull was opened, the bone cleaned, and a thin cannula (brain infusion kit 3, Alzet, Cupertino, CA, USA) linked to mini osmotic pumps (Alzet 1002) filled with (a) 0.1 M phosphate-buffered saline (PBS; vehicle), (b) mouse IFN-α (250 IU/day in 0.1 M PBS; Sigma-Aldrich, Deisenhofen, Germany), (c) poly(I:C) (1 μg/day in 0.1 M PBS; InvivoGen, San Diego, CA, USA), or (d) IFN-α (as above) and poly(I:C) (as above) implanted into the left ventricle 0.2 mm anterior and 0.9 mm lateral to bregma, as described before [4, 13] (*n* = 10 mice/group, *n* = 5/ group used for immunohistochemistry, and *n* = 5/group for Golgi-Cox staining and Western blotting). The miniosmotic pump was positioned on the back of the mice. Buprenorphine (0.1 mg/kg; Reckitt Benckiser, Slough, UK) was intraperitoneally administered as analgetic. The miniosmotic pump was left in place for 14 days until sacrifice. For animal sacrifice, mice were anesthetized with 100 mg/kg ketamine and 16 mg/kg xylazine and transcardially perfused with 4% paraformaldehyde (PFA) dissolved in 0.1 M phosphate-buffered saline (PBS) (for immunohistochemistry) or normal saline (for Golgi-Cox staining and Western blotting). PFA-fixed brains were postfixed overnight in 4% PFA and dehydrated overnight in 30% sucrose. Brains were frozen in dry ice until use. No dropouts due to pump implantation were noted. No intracerebral bleedings and no overt infections around the needle tracks were found. All animals included were employed in the data analysis.

### Dendritic Spine Quantification Ex Vivo Using Golgi-Cox Staining

To quantify dendritic spine densities ex vivo, Golgi-Cox staining was performed using the FD Rapid GolgiStain™ kit (PK401A; FD Neurotechnologies, Columbia, MD, USA). Briefly, mice were anesthetized and decapitated, and brains were processed following manufacturer’s instructions as before [14]. Coronal sections of 150 μm thickness were prepared on a cryostat starting at 0.7 mm caudal to bregma. Imaging was performed using a Zeiss AxioObserver.Z1 Inverted Microscope under a × 63 objective. Z-stacks of 90–120 μm thickness at 0.5 μm intervals were prepared. At least 10 randomly selected neurons per mouse were evaluated, of which 2 apical and 2 basal dendrites each were assessed in the CA1 region and dentate gyrus for a total of 5 mice per group. Results were expressed as dendritic spines/10 μm.

### Primary Hippocampal Neuronal Cell Cultures

Brains obtained from E15 embryos or P1–5 pups were dissected on ice cold Hank’s balanced salt solution (HBBS) buffer. The meninges were removed, and the brains were cut through the midline to expose the hippocampi, which were removed using a fine forceps and incubated in HBSS containing 0.25% trypsin (15090–046; Gibco, Schwerte, Germany) and 60 U/mL DNAse-I (D5025; Sigma) for 25 min at 37 °C. Trypsinization was stopped by addition of 5% fetal bovine serum (FBS; Invitrogen, Waltham, MA, USA) diluted in Neurobasal medium. The tissue was mechanically dissociated by repeated pipetting. Cells were seeded at a density of 7.5 × 10^4^ cells/mL on coverslips coated with 50 μg/mL poly-ornithine (P4957; Sigma) and 20 μg/mL laminin-entactin (Corning, New York, NY, USA). Neurons were incubated in Neurobasal medium containing 2% B27 (Invitrogen), glutamine (20 mM; Invitrogen), and PenStrep antibiotic mix (Invitrogen). Half of the medium was refreshed every 4–5 days. Neurons from P1–5 mice were transfected at 7 days in vitro (DIV) with a pEGFP-N1 plasmid using an optimized calcium phosphate precipitation method [15]. All cells were maintained for 12–14 DIV before initiating experiments. To study the effects of IFN-α and poly(I:C) on neuronal morphology, synapse formation, and depolarization in vitro, IFN-α (100 IU/mL), poly(I:C) (1 μg/mL), or IFN-α (100 IU/mL) and poly(I:C) (1 μg/mL) dissolved in Neurobasal medium were administered over 24 h. A subset of cells were additionally treated with K^+^ channel blocker 4-aminopyridine (4-AP; 2.5 mM; Tocris Bioscience, Wiesbaden-Nordenstadt, Germany) [16] for 1 h before protein extraction or fixation for immunocytochemistry studies.

### Immunocytochemistry

Twenty-micrometer thick coronal cryostat sections obtained from PFA-fixed animals were collected at 100 μm intervals. Primary neuronal cultures were fixed in 4% PFA in 0.1 M PBS (for dendritic spine quantification) or methanol (for synapse analyses). Sections or cells were washed, blocked with 5% normal donkey serum (NDS) in 0.1 M PBS-T, and incubated overnight with chicken anti-GFP (1:5000, Abcam), guinea-pig anti-vesicular glutamate transporter (VGLUT)-1 (1:800; Synaptic Systems, Göttingen, Germany), rabbit anti-postsynaptic density protein (PSD)-95 (1:800; Cell Signalling, Danvers, MA, USA), mouse anti-total α-amino-3-hydroxy-5-methyl-4-isoxazolepropionic acid receptor-1 (AMPAR1) (1:800; Synaptic Systems), mouse anti-neurofilament (1:500; Thermo Fisher, Waltham, MA, USA), rat anti-myelin basic protein (1:500; Abcam, Cambridge, MA, USA), or rabbit anti-BDNF (1:400; ab108319, Abcam) antibodies that were detected for 1 h at room temperature with goat anti-chicken Alexa-488 (1:500; Invitrogen), donkey anti-guinea-pig Alexa-647 (1:500; Invitrogen), donkey anti-mouse Alexa-594 (1:500; Invitrogen), donkey anti-rabbit Alexa-488 (1:500; Jackson Immunoresearch), or donkey anti-rat Alexa-594 antibodies dissolved in 0.1 M PBS-T containing 1% NDS. Cells were then washed in 0.1 M PBS and mounted with Prolong Gold medium containing 4′,6-diamidino-2-phenylindole (DAPI) (Invitrogen). For detection of DNA-fragmented neurons, neurons were labeled with a terminal deoxynucleotidyl transferase-mediated fluorescein-dUTP nick end labeling (TUNEL) kit (In Situ Cell Death Detection; Roche, Mannheim, Germany) following manufacturer’s instructions. In these studies, sections from mice exposed to middle cerebral artery occlusion for 30 min followed by 24 h reperfusion were stained as positive controls for TUNEL staining (not shown).

### Dendritic Spine Quantification In Vitro by GFP Immunocytochemistry

Ten-micrometer thick Z-stacks were prepared at 0.1 μm intervals from neurons labeled with anti-GFP antibody using the same microscope and Apotome correction function as above. Maximal projections were digitally obtained using Image J. Images of two primary dendrites per neuron from a total of 45 neurons per treatment were obtained from three independent experiments. Both for ex vivo and in vitro analyses, a section of 20–30 μm length of the primary dendrite was analyzed using the plugin Simple Neurite Tracer from Image J. Results were expressed as dendritic spines/10 μm.

### Synaptic Density Quantification

Synaptic density quantifications were performed as described previously [17] using our in-house Synapse Counter plugin for ImageJ (freely available at https://github.com/SynPuCo/SynapseCounter). Briefly, the structurally complete glutamatergic synapses were identified by the spatial overlap of presynaptic marker VGLUT1 and postsynaptic markers PSD95. For each condition, 56 micrographs were obtained (*n* = 4) using the Carl Zeiss LSM 710 confocal microscope using the alpha Plan-Apochromat × 100 oil objective. The densities of synaptic puncta were quantified in 66.5 × 66.5 μm regions containing a single neuron perikaryon.

### Western Blots

For protein extraction, hippocampi were quickly dissected, homogenized, and lysed in 1% NP-40 buffer containing 50 mmol/L Tris-HCl and 150 mmol/L NaCl (pH 7.4) supplemented with 5% protease inhibitor cocktail and 1% phosphatase inhibitor cocktail-2. Tissues were centrifuged at 13,000 rpm at 4 °C for 15 min. Supernatants were collected and immediately frozen at − 80 °C until use. Proteins from cell cultures were extracted using the Trizol (Thermo Fisher) method following the manufacturer’s instructions and frozen at − 80 °C until use. Protein concentration was estimated by the Bradford method (Quick Start Bradford Protein Assay; Bio-Rad, Hercules, CA, USA). Ten microgram of samples (for ex vivo experiments) and 10–50 μg samples (for in vitro experiments) were loaded on 10% sodium dodecyl sulfate polyacrylamide gels. After electrophoretic separation, proteins were transferred to PVDF membranes. Membranes were blocked with 5% nonfat-dried milk (Sigma-Aldrich) dissolved in 0.1 M PBS-T for 1 h at room temperature, washed and incubated overnight at 4 °C with rabbit anti-VGLUT1 (1:2000; Synaptic Systems), rabbit anti-VGLUT2 (1:1000; Synaptic Systems), mouse anti-PSD95 (1:1000; Synaptic Systems), mouse anti-total AMPAR1; 1:1000; Synaptic Systems), rabbit anti-phospho-Ser^831^-AMPAR1 (1:1000; Synaptic Systems), rabbit anti-total TrkB (1:1000; Cell Signaling), rabbit anti-phospho-TrkB (1:1000; Abcam), rabbit anti cAMP-response element containing binding protein (CREB) (1:1000; Millipore, Temecula, CA, USA), mouse anti-phosphorylated CREB (1:1000; Millipore), mouse anti-synaptotagmin (1:1000; Synaptic Systems), goat anti-excitatory amino acid transporter-2 (EAAT2) (1:1000; Santa Cruz Biotechnology, Dallas, TX, USA), and rabbit anti-tubulin (1:5000; Synaptic Systems) antibodies. Secondary peroxidase-coupled goat anti-mouse (1:5000; Santa Cruz, Dallas, TX, USA) or donkey anti-rabbit (1:5000; Jackson Immunoresearch, Cambridgeshire, UK) antibodies were incubated for 1 h at room temperature. For calculating phosphorylation ratios, phosphorylated and total forms of proteins were detected on different membranes and normalized against tubulin levels. Blots were developed using Amersham ECL Prime Western Blotting Detection Reagent (Life Sciences, Waltham, MA, USA) and scanned using a digital enhanced chemiluminescence (ECL) detection device (Thermo Fisher). Samples from 5 animals per group were analyzed in triplicate.

### Patch Clamping

Hippocampal neurons at 12–14 DIV were either stimulated overnight with IFN-α (100 IU/mL), poly(I:C) (1 μg/mL), or IFN-α (100 IU/mL) plus poly(I:C) (1 μg/mL). Prior to recording, culture medium was replaced with an external solution containing 1.2 mM MgCl_2_, 10 mM HEPES, 10 mM glucose, 1.5 mM CaCl_2_, 2.5 mM KCl, and 145 mM NaCl (pH 7.4). The pipette solution contained 140 mM KCl, 1 mM CaCl_2_·2H_2_O, 4 mM MgCl_2_, 10 mM HEPES, 0.4 mM Na_2_-GTP, 4 mM Mg-ATP, and 10 mM EGTA (pH 7.3). Microelectrodes of 1.5 mm thin-walled borosilicate glass (World Precision Instruments, Friedberg, Germany) were pulled with a DMZ-Universal Puller (Zeitz-Instruments, Planegg, Germany) and polished to a final resistance of 3–4 MΩ. A giga-Ω seal was established between the pipette and membrane. The resting membrane potential was determined by injection of zero current after establishing the whole cell configuration. Current clamp recordings were acquired in the whole cell configuration using the patch clamp method. For the experiments, current was injected to hold neurons at a membrane potential of − 65 mV, which resembles resting potential of hippocampal neurons. To determine action potential threshold, current clamp was used, and neurons were stimulated with a stepper protocol by increasing current steps until action potentials were elicited. Action potential frequency was recorded after injecting currents of two-fold the action potential threshold. Voltage clamp recordings were performed in whole cell configuration, and neurons were clamped at − 65 mV. Data was acquired with an Axopatch 200B amplifier (Molecular Devices, Biberach, Germany) with pClamp software 10.6 and analyzed with Clampfit software 10.6 (Molecular Devices). The signal was continuously sampled at a frequency of 5 kHz and filtered at 2.0 kHz.

### Statistics

All ex vivo data sets and in vitro data sets from depolarized neurons or patch clamp studies were analyzed by one-way analysis of variance (ANOVA) and Tukey’s post hoc test. For in vitro morphological data sets, two-way ANOVA with the between group factors treatment (vehicle, IFN-α, poly (I:C) and IFN-α/poly(I:C)) and depolarization (Non-depolarized, depolarized) followed by Tukey’s post hoc test were also used. *p* < 0.05 was considered significant.

## Results

### IFN-α and Poly(I:C) Delivery Reduce Dendritic Spine Density in the CA1 Region Ex Vivo

**Fig. 1:**
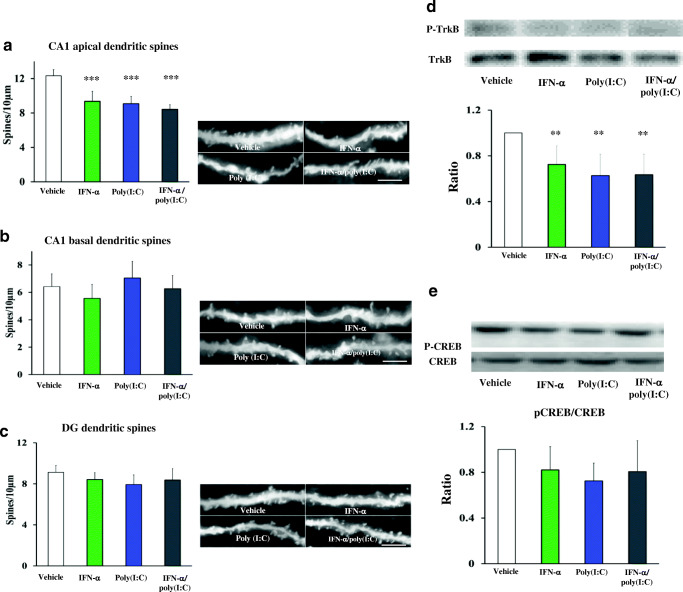
Delivery of IFN-α and poly(I:C) reduces apical dendritic spine density of CA1 neurons and decreases TrkB signaling ex vivo. Spine density of **a** CA1 apical dendrites, **b** CA1 basal dendrites, and **c** dentate gyrus dendrites evaluated by Golgi-Cox staining in mice exposed to vehicle, IFN-α (250 IU/day), poly(I:C) (1 μg/day), or combined IFN-α and poly(I:C). Phosphorylation level of **d** TrkB and **e** CREB in the hippocampus of the same mice evaluated by Western blots. For the latter analyses, Western blots for phosphorylated and total TrkB and CREB were performed, and expression levels were related to each other. No significant differences of CREB phosphorylation were found between groups. Data are means ± SD, analyzed by one-way ANOVA followed by Tukey’s post-hoc tests (≥ 10 randomly selected neurons per mouse evaluated in A, of which 2 apical and 2 basal dendrites each were examined in *n* = 5 mice/ group; tissue samples of *n* = 5 mice/group separately evaluated in B as triplicates). **p < 0.01, ***p < 0.001 compared with vehicle-treated animals. Scale bars = 5 μm.

To gain insight into neuronal plasticity changes in the IFN-α- and poly(I:C)-induced depression model, we first analyzed the dendritic spine density of neurons within the hippocampal CA1 region and dentate gyrus using Golgi-Cox stainings ex vivo (Fig. 1a). We observed that IFN-α, poly(I:C) or combined IFN-α and poly(I:C) delivery significantly reduced the density of apical dendritic spines in the CA1 region (Fig. 1a) [F(3,15) = 4; *p* = 0.028], but not of basal dendritic spines in the CA1 region (Fig. 1b) or dendritic spines in the dentate gyrus (Fig. 1c). The overall length of dendritic spines was unchanged (Suppl. Fig. 1A–C). Notably, the three treatments influenced dendritic spine density to a very similar extent (Fig. 1a). We previously reported that IFN-α and poly(I:C) additively induce depressive symptoms in mice [4]. Western blot analysis revealed that phosphorylation of the BDNF receptor TrkB (Fig. 1d) but not the transcription factor CREB (Fig. 1e), which both mediate neuronal plasticity, was significantly reduced by IFN-α poly(I:C) or combined IFN-α and poly(I:C) delivery [F(3,12) = 3.88; *p* = 0.038]. Immunohistochemical analyses showed a reduction of the number of BDNF expressing neurons in the hippocampal CA1 region [F(3,27) = 4.73; *p* = 0.010], but not DG [F(3,30) = 1.13; *p* = 0.353] after poly(I:C) exposure, which was not observed in the combined IFN-α and poly(I:C) delivery group (Suppl. Fig. 2). We did not find evidence of neuronal degeneration or demyelination in the hippocampus (Suppl. Figs. 3 and 4).

### IFN-α and Poly(I:C) Differentially Regulate Pre- and Postsynaptic Proteins of Glutamatergic Synapses Ex Vivo

**Fig. 2:**
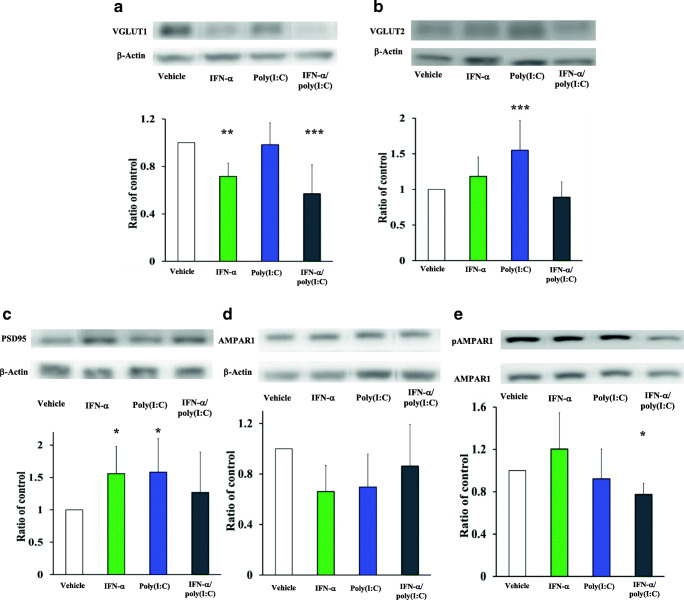
IFN-α and poly(I:C) differentially regulate pre- and postsynaptic proteins of glutamatergic synapses ex vivo. Abundance of **a** presynaptic glutamatergic proteins VGLUT1 and **b** VGLUT2, abundance of postsynaptic proteins **c** PSD95 and **d** AMPAR1, as well as **e** phosphorylation level of AMPAR1 at the Ser^831^ residue evaluated by Western blots in the hippocampus of mice exposed to vehicle, IFN-α (250 IU/day), poly(I:C) (1 μg/day), or IFN-α and poly(I:C) (as before). Note that VGLUT1 abundance is reduced by IFN-α or combined IFN-α and poly(I:C) delivery. VGLUT2 is increased by poly(I:C), and PSD95 abundance is increased by IFN-α or poly(I:C). Whereas total AMPAR1 abundance is unchanged, Ser^831^AMPAR1 phosphorylation is reduced by combined IFN-α and poly(I:C) delivery. Data are means ± SD, analyzed by one-way ANOVA followed by Tukey’s post hoc tests (tissue samples of *n* = 5 mice/group evaluated as triplicates). *p < 0.05, **p < 0.001, ***p < 0.001 compared with vehicle

Since dendritic spines are sites of major synaptic input, we next evaluated the abundance of pre- and postsynaptic proteins of glutamatergic synapses by Western blots. Our results showed that levels of presynaptic protein VGLUT1 were significantly reduced by IFN-α or combined IFN-α and poly(I:C) delivery (Fig. 2a; Suppl. Fig. 5) [F(3,27) = 12.77; *p* < 0.001], while VGLUT2 was increased only by poly(I:C), but not combined IFN-α and poly(I:C) delivery (Fig. 2b; Suppl. Fig. 5) [F(3,36) = 9.77; *p* < 0.001]. The postsynaptic protein PSD95 was increased by IFN-α or poly(I:C), but not by combined IFN-α and poly(I:C) delivery (Fig. 2c; Suppl. Fig. 5) [F(3,36) = 3.60; *p* = 0.022]. Importantly, the total level of the postsynaptic protein AMPAR1 was unchanged by IFN-α and poly(I:C), both when administered alone or in combination (Fig. 2d; Suppl. Fig. 5D), whereas Ser^831^AMPAR1 phosphorylation relative to total AMPAR1 level was significantly reduced by combined IFN-α and poly(I:C) delivery (Fig. 2e) [F(3,31) = 4.94; *p* = 0.01]. Phosphorylation of AMPAR1 is a necessary step for the insertion of the AMPAR1 in the postsynaptic membrane that is required for fast neurotransmission [18]. The presynaptic Ca^2+^ sensor synaptotagmin-1, which is required for synaptic vesicle fusion with the presynaptic membrane, and glial glutamate transporter EAAT2, which removes glutamate from or returns glutamate to the synaptic cleft, were not altered by IFN-α and poly(I:C), neither when administered alone nor in combination (Suppl. Fig. 6A, B). Synaptotagmin-1 had been shown to remain unchanged after prenatal poly(I:C) delivery in the past [19].

### Morphological Effects of IFN-α and Poly(I:C) Stimulation Are Recapitulated In Vitro

**Fig. 3:**
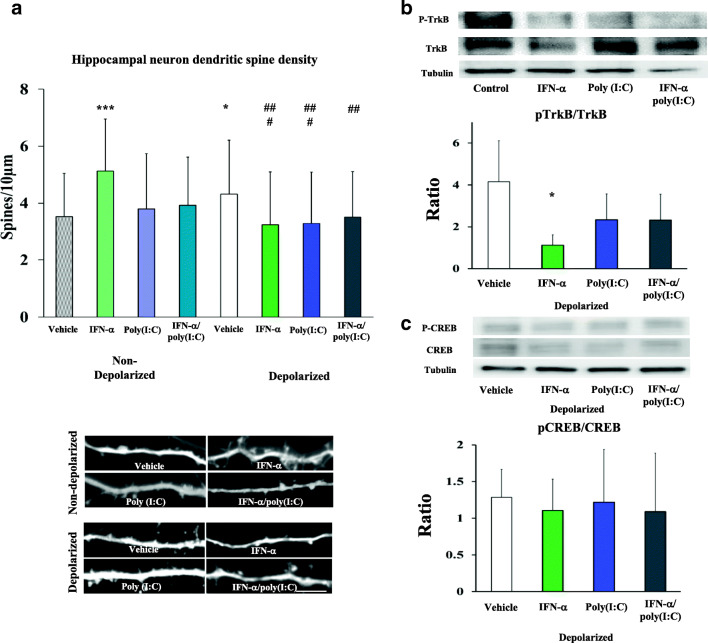
Delivery of IFN-α and poly(I:C) reduces dendritic spine density of primary hippocampal neurons and decreases TrkB signaling in vitro in a neuronal activity-dependent way. **a** Dendritic spine density of primary hippocampal neurons that were either non-depolarized or had been depolarized by 4-AP (2.5 mM) at 14 days in vitro (DIV) and had been exposed to vehicle, IFN-α (100 IU/mL), poly(I:C) (1 μg/mL), or IFN-α and poly(I:C) (as before). Neuronal depolarization with 4-AP increased dendritic spine density compared with non-depolarized vehicle conditions. IFN-α and poly(I:C) reduced dendritic spine density in depolarized, but not in non-depolarized cells both when administered alone and in combination with each other. Abundance of **b** TrkB phosphorylation and **c** CREB phosphorylation in depolarized primary hippocampal neurons (2.5 mM 4-AP) exposed to vehicle conditions, IFN-α 100 (IU/mL), poly(I:C) (1 μg/mL), or IFN-α and poly(I:C) (as before) at 14 DIV evaluated by Western blots. TrkB phosphorylation was significantly reduced by IFN-α, whereas CREB phosphorylation was not influenced by IFN-α and poly(I:C). Data are mean ± SD, analyzed by one-way ANOVA followed by Tukey’s post hoc tests (*n* = 3 experiments with ≥ 15 randomly selected neurons per experiment evaluated in A, of which 2 dendrites each were examined; *n* = 3 experiments evaluated in B as triplicates). *p < 0.05, ***p < 0.001 compared with non-depolarized vehicle, ^##^*p* < 0.01, ^###^*p* < 0.001 compared with depolarized vehicle. Scale bar = 5 μm

To further evaluate effects of IFN-α or TLR3 activation on synaptic function, we examined morphological responses of primary hippocampal neurons exposed to IFN-α and poly(I:C) at 14 DIV. One significant difference between the adult hippocampus ex vivo and 14 DIV hippocampal neurons in vitro is the presence of basal neuronal activity. To address this issue, we depolarized neurons with 4-AP (2.5 mM) 1 h before fixation and administered IFN-α and poly(I:C). We observed a tendency towards a significant main effect for treatments [F(3,433) = 2.6; *p* = 0.051], a significant main effect for depolarization [F(1,433) = 8.98; *p* = 0.003], and a significant interaction between treatments and depolarization [F(3,433) = 10.4; *p* < 0.001]. Post hoc analysis showed that IFN-α delivery significantly increased the density of dendritic spines under non-depolarized conditions, while poly(I:C) and combined IFN-α and poly(I:C) delivery had no effect (Fig. 3a). Depolarization significantly increased the density of dendritic spines in vehicle-treated cells compared with non-depolarized vehicle conditions (Fig. 3a). In response to depolarization, IFN-α and poly(I:C), both when applied alone and in combination, significantly reduced the density of dendritic spines (Fig. 3a). Again, all three treatments influenced dendritic spine density to a very similar extent. Hence, the morphological responses of depolarized neurons resembled the effects seen ex vivo. Western blots revealed that, under depolarized conditions, TrkB phosphorylation was significantly reduced by IFN-α, whereas TrkB phosphorylation following poly(I:C) and combined IFN-α and poly(I:C) delivery did not differ from the depolarized vehicle conditions (Fig. 3b) [F(3,14) = 3.56; *p* = 0.042]. We did not observe any effect of IFN-α and poly(I:C) on CREB phosphorylation, neither when applied alone or in combination (Fig. 3c). Hence, the underlying mechanisms of action are apparently similar ex vivo and in vitro, indicating a role of BDNF signaling that occurs in a neuronal activity-dependent manner.

### IFN-α and Poly(I:C) Dysregulate Glutamatergic Synapses In Vitro

**Fig. 4:**
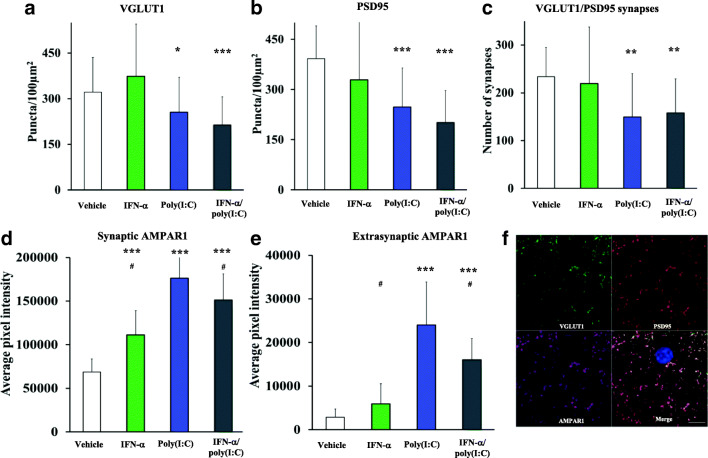
Loss of glutamatergic synapses upon IFN-α and poly(I:C) treatment in vitro. Glutamatergic synapses were evaluated after exposure of primary hippocampal neurons to vehicle, IFN-α (100 IU/mL), poly(I:C) (1 μg/mL), or IFN-α and poly(I:C) (as before) at 14 DIV, followed by 1 h 4-AP-induced (2.5 mM) neuronal depolarization for 1 h. Abundance of **a** VGLUT1 and **b** PSD95 was reduced by poly(I:C) and combined IFN-α and poly(I:C) delivery causing an overall significant reduction of **c** glutamatergic VGLUT1/PSD95 synapses. **d** Intrasynaptic and **e** extrasynaptic AMPAR1 expression analysis showed a compensatory increase induced by poly(I:C) that was counteracted by IFN-α in the combined stimulation condition. Representative images of analyzed synapses are shown in **f** (DAPI counterstaining in blue). Data are mean ± SD, analyzed by one-way ANOVA followed by Tukey’s post hoc tests (*n* = 4 independent experiments with a total of 56 cells/group). *p < 0.05, **p < 0.01, ***p < 0.001 compared with vehicle/^#^*p* < 0.05 compared with poly(I:C). Scale bar = 5 μm

To determine whether glutamatergic synapses were also disrupted in vitro, we examined the expression of the presynaptic protein VGLUT1 and the postsynaptic proteins PSD95 and AMPAR1 in hippocampal primary neurons depolarized by 4-AP by immunocytochemistry. Here, we observed a reduction of the number of VGLUT1 (Fig. 4a) [F(3,112) = 8.74; *p* = 0.001] and PSD95 (Fig. 4b) [F(3,108) = 2.68; *p* < 0.001] puncta by poly(I:C) and combined IFN-α and poly(I:C) delivery. Consequently, the number of glutamatergic synapses as defined by VGLUT1/PSD95 colocalization was reduced (Fig. 4c) [F(3,106) = 6.08; *p* = 0.001]. In contrast, we observed that AMPAR1 expression associated to VGLUT1/PSD95 synapses (e.g., synaptic AMPAR1) showed a compensatory increase induced by poly(I:C) and combined IFN-α and poly(I:C) delivery [F(3,112) = 90.07; *p* < 0.001] (Fig. 4d). Extrasynaptic AMPAR1 expression also showed a compensatory increase induced by poly(I:C) that was partly attenuated by IFN-α (Fig. 4e) [F(3,112) = 70.91; *p* < 0.001]. This is in contrast to ex vivo measurements where we observed no change of AMPAR1 levels. These data suggest that AMPAR1 trafficking may also be compromised in vitro by inflammatory conditions. The reduction of PSD95 and consequent increase of synaptic and extrasynaptic AMPAR1 suggest a dissociation of AMPAR1 and PSD95 that could impair AMPAR1 efficacy and neuronal excitability. AMPAR1 phosphorylation was tendentially reduced by poly(I:C) but not combined IFN-α and poly(I:C) delivery (Suppl. Fig. 7) [F(3,11) = 2.38; *p* = 0.145].

### IFN-α and Poly(I:C) Decrease Neuronal Excitability In Vitro

**Fig. 5:**
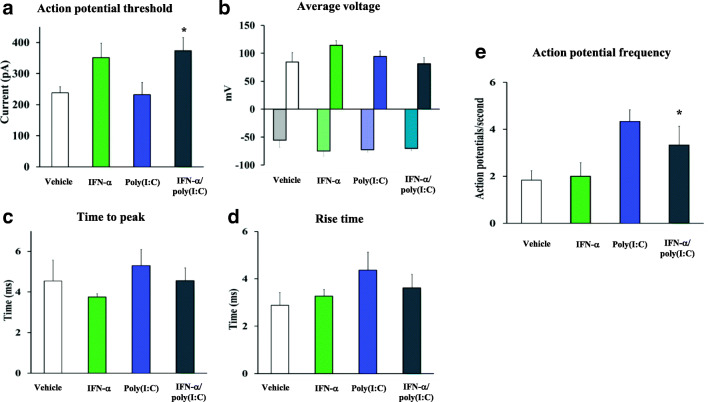
IFN-α and poly(I:C) impair neuronal excitability. Neuronal excitability was evaluated by patch clamp recording of neurons incubated overnight with vehicle, IFN-α (100 IU/mL), poly(I:C) (1 μg/mL), or IFN-α and poly(I:C) (as before). IFN-α induced a significant increase in **a** action potential threshold only in the presence of poly(I:C). No alterations were observed for **b** average voltage, **c** time to peak, or **d** rise time of the action potential. Poly(I:C) significantly increased the action potential frequency, and IFN-α partially reverted this effect when combined with poly(I:C). Data are mean ± SEM, analyzed by one-way ANOVA, followed by Tukey’s post hoc tests (*n* = 5–8 neurons/group). *p < 0.05 compared to vehicle

Finally, we asked whether neuronal excitability would be altered by IFN-α or poly(I:C) in 14 DIV neurons. Patch clamp recordings revealed that IFN-α increased the action potential threshold. However, this effect was only statistically significant when IFN-α was used in combination with poly(I:C) (Fig. 5a) [F(3,21) = 3.71; *p* = 0.027]. IFN-α and poly(I:C) did not induce significant changes to average voltage (Fig. 5b), time to peak (Fig. 5c), and rise time (Fig. 5d) of the action potential when applied alone or in combination. We observed that the frequency of action potentials was significantly increased by poly(I:C) although this effect was partially antagonized by costimulation with IFN-α (Fig. 5e) [F(3,18) = 3.96; *p* = 0.025]. Since action potential threshold and frequency are dependent on Na^+^ channels [20], we conclude that these results suggest that IFN-α and poly(I:C) impair neuronal excitability by reducing Na^+^ channel functionality.

## Discussion

By combining ex vivo and in vitro studies, we report here morphological, biochemical, and electrophysiological correlates of reduced dendritic and synaptic plasticity in the CA1 region of the hippocampus and dentate gyrus in a model of IFN-α and TLR3 activation-induced depression in mice which we previously characterized on the behavioral level [4]. While previous studies evaluated the effect of IFN-α or poly(I:C) on behavior and inflammation when administered alone, we stringently characterized the independent and combined effects of both stimuli on several aspects of hippocampal neuronal plasticity. Stimulation with IFN-α and poly(I:C) reduced CA1 apical dendritic spine density in association with reduced VGLUT1 levels and TrkB phosphorylation and reduced BDNF expression in the hippocampal CA1 region ex vivo. Notably, while AMPAR1 and PSD95 levels were stable, Ser^831^AMPAR1 phosphorylation was mildly reduced ex vivo but not in vitro by combined IFN-α and poly(I:C) delivery. Decreased dendritic spine density and reduced levels of VGLUT1 were observed in vitro only after neuronal depolarization was induced by 4-AP. Remarkably, PSD95 was reduced, and AMPAR1 was increased in vitro under neuronal depolarization conditions. This indicates that the presynaptic compartment (e.g., VGLUT1) is particularly sensitive to IFN-α or poly(I:C) stimulation, therefore suggesting that presynaptic mechanisms of glutamate release may play a primary role during the early stages of immune-induced depression as compared with the postsynaptic compartment. The observed differences between ex vivo and in vitro conditions may be explained by the absence of glial and microglial inflammatory responses in vitro that we previously characterized ex vivo [4]. Patch clamp studies revealed that IFN-α increased the action potential threshold of primary hippocampal neurons, while poly(I:C) increased the frequency of action potentials. Hence, our studies suggest that disruption of glutamatergic synapses by IFN-α and poly(I:C) leads to insufficient neurotrophic support through BDNF-TrkB signaling, resulting in reduced dendritic spine density and impaired electrophysiological activity as hallmarks of depression (Suppl. Fig. 8).

The delivery of inflammatory cytokines and immune stimulants is widely used to study depression pathogenesis [21]. Previous studies already suggested that inflammation may negatively influence BDNF signaling, thus limiting neuronal plasticity in the hippocampus, prefrontal cortex, amygdala, and raphe nuclei and causing the emergence of depressive-like behavior [21] although behavioral, structural, molecular, and electrophysiological data remain poorly integrated. IFN-α was shown to decrease BDNF synthesis and primary hippocampal neuronal branching in vitro by mechanisms involving NMDA receptor signaling [22] and to reduce hippocampal neurogenesis and cause fatigue and immobility in tail suspension and forced swim tests in mice [23]. Poly(I:C) has been reported to decrease cortical neuron branching by promoting cytokine release from monocytes in vivo [10, 12], reduce pre- and postsynaptic protein content, interfere with memory formation, reduce neuronal excitability, and render the brain more susceptible to stress during adulthood [24, 25]. Patients suffering from IFN-α therapy-related depression show negative regulation of vast signaling pathways relevant for neuronal plasticity including extracellular axonal guiding molecules and subcellular signaling deficiencies related to cytoskeleton dynamics and neuronal morphology (e.g., ephrins and rho GTPases) [26]. Our data expands previous results by comparing the individual effects of IFN-α and poly(I:C) to the combined condition. Although we did not evaluate behavioral correlates of depression in the present study, we demonstrated in an earlier study [4] that combined IFN-α and poly(I:C) were necessary to induce significant depression-like symptoms. In the present study, both IFN-α and poly(I:C) reduced dendritic and synaptic plasticity when administered alone, yet with diverse actions on different glutamatergic markers that were sometimes antagonistic. For example, IFN-α reversed the upregulation of VGLUT2 by poly(I:C) ex vivo, attenuated the upregulation of AMPAR1 by poly(I:C) in vitro, and attenuated the increase of action potential frequency by poly(I:C) in vitro. Our data suggest that both IFN-α and poly(I:C) reduce dendritic and synaptic plasticity and that reduced plasticity may predispose to the development of depression-like symptoms when signaling processes in the pre- and postsynaptic membranes are simultaneously modified. This may explain the additive influence of both stimuli on the manifestation of depression-like symptoms [4]. Of note, IFN-α and poly(I:C) are sometimes delivered intraperitoneally over longer periods or at higher doses to achieve depressive-like behaviors [27]. Apparently, intraventricular delivery results in accelerated molecular, structural, and behavioral responses.

Glutamatergic transmission has been proposed as a key part of the etiology of mood disorders [8]. IFN-α-treated HCV patients showed increased ratios of glutamine to glutamate in the cerebral cortex (indicative of low cortical glutamate), which positively correlated with depression and anxiety [28]. It was recently observed that glutamatergic activation of the infralimbic cortex by glutamate transporter-1 blockade or AMPA microinfusion reduced depression-like behavior by promoting serotonin release from the raphe nuclei in rats [29], which may partly explain how serotonin reuptake inhibitors reduce IFN-α-mediated depression [30]. Importantly, another study found a correlation of VGLUT1 downregulation in the cerebral cortex and depression in postmortem human samples [31]. Inflammatory processes therefore may influence the emergence of depression not only by inhibiting physiological neuronal sprouting or synaptic pruning but also by limiting glutamate availability within the synapse. Indeed, IL-1, TNF-α, IFN-γ, and poly(I:C) inhibit astrocytic glutamate uptake by deactivating the glutamate transporter EAAT1, thus modulating NMDA spontaneous currents in hippocampal slices [32–34]. Our data further adds that VGLUT1 is reduced by either IFN-α or poly(I:C), thus probably reducing glutamate availability within the synaptic cleft given that VGLUT1 elimination reduces the synaptic vesicle pool and also decreases neuronal excitability [35]. Importantly, acute IFN-α stimulation of hippocampal slices reduced miniature excitatory postsynaptic excitatory currents [36], which is consistent with a reduction of presynaptic glutamate release as indicated by our analyses of VGLUT1 expression.

Given the fact that glutamate release regulates BDNF synthesis and TrkB activation through CREB phosphorylation [7], a reduction of glutamate release as a consequence of reduced VGLUT1 may provide a stringent explanation for reduced TrkB signaling in our study. Interestingly, type I IFNs and TLR3 activation can independently modulate the expression of BDNF and TrkB. IFN-β reduced neuronal differentiation of primary cortical cells through downregulation of BDNF and TrkB via a JAK/STAT-1-dependent mechanism [37]. Intraperitoneal administration of poly(I:C) caused a very fast reduction of BDNF and TrkB mRNA in the frontal cortex and hippocampus [38]. Depression scores were negatively associated with serum BDNF levels in IFN-α−treated HCV patients [39]. TrkB signaling and electrophysiological activity are tightly interrelated in animal models of mood disorders [7], which is in line with our finding of reduced neuronal excitability in response to IFN-α and poly(I:C) exposure. Future studies should further characterize local plasticity responses in defined subcellular compartments (e.g., synaptosomes). In early stages of depression pathogenesis, such studies may help to identify promising therapeutic targets for new antidepressant drugs, via which the compromised neuroplasticity may be restored.

## Electronic Supplementary Material

Supplementary Fig. 1 IFN-α and poly(I:C) do not induce changes of dendritic spine length ex vivo. No significant differences were found of the length of dendritic spines in either (A) apical or (B) basal CA1 or (C) dentate gyrus neurons in Golgi-Cox stained sections from mice exposed to vehicle, IFN-α (250 IU/day), poly(I:C) (1 μg/day) or combined IFN-α and poly(I:C) (as before) delivery. Data are means ± S.D. No significant differences were noted between groups (≥10 randomly selected neurons per mouse evaluated in A, of which 2 apical and 2 basal dendrites each were examined in n = 5 mice/ group; tissue samples of n = 5 mice/ group separately evaluated in B as triplicates) (PPTX 82 kb)

Supplementary Fig. 2: Poly(I:C) reduces BDNF expression ex vivo in the hippocampus. (A) Representative images of BDNF expressing cells in the hippocampus of mice exposed to vehicle, IFN-α (250 IU/day), poly(I:C) (1 μg/day) or combined IFN-α and poly(I:C) (as before) delivery. Squares show regions of interest in the CA1 and DG regions which were quantified. Poly(I:C), but not IFN-α or combined IFN-α and poly(I:C) delivery, significantly reduced the number of BDNF expressing cells in the CA1 region (B), but not the DG (C). Data are means ± SD, analyzed by one-way ANOVA, followed by Tukey post-hoc tests (*n* = 4–5 mice/group). **p = 0.01 compared to vehicle. (PPTX 804 kb)

Supplementary Fig. 3 IFN-α and poly(I:C) do not induce neurodegeneration or demyelination ex vivo. Neurofilament and myelin basic protein images were obtained from (A) the CA1 stratum lacunosum moleculare (slm) and (B) dentate gyrus polymorph layer (po) of mice exposed to vehicle, IFN-α (250 IU/day), poly(I:C) (1 μg/day) or combined IFN-α and poly(I:C) (as before) delivery. No significant differences of neurofilament or myelin basic protein density were found in (C, D) the CA1 region or (E, F) the dentate gyrus. Scale bars = 30 μm in A, 10 μm in B. (PPTX 741 kb)

Supplementary Fig. 4 IFN-α and poly(I:C) do not induce neuronal death ex vivo. No evidence of irreversibly injured, that is, TUNEL+ neurons was found in the CA1 region or in other areas of the hippocampus of mice exposed to vehicle, IFN-α (250 IU/day), poly(I:C) (1 μg/day) or combined IFN-α and poly(I:C) (as before) delivery. DAPI counterstainings are shown in blue. Scale bar = 20 μm. (PPTX 1088 kb)

Supplementary Fig. 5 Representative Western blots for the presynaptic proteins VGLUT1 and VGLUT2 and the postsynaptic proteins PSD95, AMPAR1. Protein lysates were obtained from the hippocampus of mice exposed to vehicle, IFN-α (250 IU/day), poly(I:C) (1 μg/day) or combined IFN-α and poly(I:C) (as before) delivery. (PPTX 1213 kb)

Supplementary Fig. 6 IFN-α and poly(I:C) do not influence the presynaptic Ca^++^ sensor synaptotagmin-1 or the glutamate transporter EAAT2 ex vivo. (A) Synaptotagmin 1, which needs to be activated for synaptic vesicle fusion with the presynaptic membrane, or (B) EAAT2, which removes glutamate from or returns glutamate to the synaptic cleft, thus influencing glutamatergic transmission, were unchanged, as shown by Western blots of mice exposed to vehicle, IFN-α (250 IU/day), poly(I:C) (1 μg/day) or combined IFN-α and poly(I:C) (as before) delivery. Data are means ± S.D. No significant differences were observed between groups (*n* = 5 mice/ group evaluated as triplicates). (PPTX 196 kb)

Supplementary Fig. 7 Phosphorylation of AMPAR1 does not change in vitro in response to IFN-α and poly(I:C) exposure. Phosphorylation level of AMPAR1 of primary hippocampal neurons depolarized for 1 h by 4-AP (2.5 mM) after exposure to vehicle, IFN-α (100 IU/mL), poly(I:C) (1 μg/mL) or IFN-α and poly(I:C) (as before). No significant changes of AMPAR1 phosphorylation were noted. Data are means ± S.D. (*n* = 3 experiments evaluated as triplicates). (PPTX 68 kb)

Supplementary Fig. 8 Summary of findings. In response to IFN-α and poly(I:C) exposure, synaptic plasticity is compromised in CA1 apical dendrites. At the molecular level, reduced TrkB phosphorylation is accompanied by the decreased synthesis of the presynaptic protein VGLUT1 and postsynaptic protein PSD95. The altered synaptic plasticity is thought to contribute to IFN-α/ poly(I:C) associated depression. (PPTX 41 kb)

## References

[1] Hoyo-Becerra C, Huebener A, Trippler M, Lutterbeck M, Liu ZJ, Truebner K, Bajanowski T, Gerken G, Hermann DM, Schlaak JF (2013). Concomitant interferon alpha stimulation and TLR3 activation induces neuronal expression of depression-related genes that are elevated in the brain of suicidal persons. PLoS One.

[2] Schaefer M, Schwaiger M, Pich M, Lieb K, Heinz A (2003). Neurotransmitter changes by interferon-alpha and therapeutic implications. Pharmacopsychiatry.

[3] Hoyo-Becerra C, Schlaak JF, Hermann DM (2014). Insights from interferon-alpha-related depression for the pathogenesis of depression associated with inflammation. Brain Behav Immun.

[4] Hoyo-Becerra C, Liu Z, Yao J, Kaltwasser B, Gerken G, Hermann DM, Schlaak JF (2015). Rapid regulation of depression-associated genes in a new mouse model mimicking interferon-alpha-related depression in hepatitis C virus infection. Mol Neurobiol.

[5] Godsil BP, Kiss JP, Spedding M, Jay TM (2013). The hippocampal-prefrontal pathway: the weak link in psychiatric disorders?. Eur Neuropsychopharmacol.

[6] Lin CW, Chen CY, Cheng SJ, Hu HT, Hsueh YP (2014). Sarm1 deficiency impairs synaptic function and leads to behavioral deficits, which can be ameliorated by an mGluR allosteric modulator. Front Cell Neurosci.

[7] Tejeda GS, Diaz-Guerra M (2017) Integral characterization of defective BDNF/TrkB signalling in neurological and psychiatric disorders leads the way to new therapies. Int J Mol Sci 18(2). 10.3390/ijms18020268 10.3390/ijms18020268 PMC5343804 28134845

[8] Sanacora G, Treccani G, Popoli M (2012). Towards a glutamate hypothesis of depression: An emerging frontier of neuropsychopharmacology for mood disorders. Neuropharmacology.

[9] Datwani A, McConnell MJ, Kanold PO, Micheva KD, Busse B, Shamloo M, Smith SJ, Shatz CJ (2009). Classical MHCI molecules regulate retinogeniculate refinement and limit ocular dominance plasticity. Neuron.

[10] Chen CY, Liu HY, Hsueh YP (2017). TLR3 downregulates expression of schizophrenia gene Disc1 via MYD88 to control neuronal morphology. EMBO Rep.

[11] Hung YF, Chen CY, Shih YC, Liu HY, Huang CM, Hsueh YP (2018). Endosomal TLR3, TLR7, and TLR8 control neuronal morphology through different transcriptional programs. J Cell Biol.

[12] Garre JM, Silva HM, Lafaille JJ, Yang G (2017). CX3CR1(+) monocytes modulate learning and learning-dependent dendritic spine remodeling via TNF-alpha. Nat Med.

[13] Fitzgerald PJ, Yen JY, Watson BO (2019). Stress-sensitive antidepressant-like effects of ketamine in the mouse forced swim test. PLoS One.

[14] Herring A, Munster Y, Akkaya T, Moghaddam S, Deinsberger K, Meyer J, Zahel J, Sanchez-Mendoza E, Wang Y, Hermann DM, Arzberger T, Teuber-Hanselmann S, Keyvani K (2016). Kallikrein-8 inhibition attenuates Alzheimer's disease pathology in mice. Alzheimers Dement.

[15] Kononenko NL, Diril MK, Puchkov D, Kintscher M, Koo SJ, Pfuhl G, Winter Y, Wienisch M, Klingauf J, Breustedt J, Schmitz D, Maritzen T, Haucke V (2013). Compromised fidelity of endocytic synaptic vesicle protein sorting in the absence of stonin 2. Proc Natl Acad Sci U S A.

[16] Kaufman AM, Milnerwood AJ, Sepers MD, Coquinco A, She K, Wang L, Lee H, Craig AM, Cynader M, Raymond LA (2012). Opposing roles of synaptic and extrasynaptic NMDA receptor signaling in cocultured striatal and cortical neurons. J Neurosci.

[17] Dzyubenko E, Gottschling C, Faissner A (2016). Neuron-glia interactions in neural plasticity: contributions of neural extracellular matrix and perineuronal nets. Neural Plast.

[18] Jenkins MA, Traynelis SF (2012). PKC phosphorylates GluA1-Ser831 to enhance AMPA receptor conductance. Channels (Austin).

[19] Forrest CM, Khalil OS, Pisar M, Smith RA, Darlington LG, Stone TW (2012). Prenatal activation of toll-like receptors-3 by administration of the viral mimetic poly(I:C) changes synaptic proteins, N-methyl-D-aspartate receptors and neurogenesis markers in offspring. Molecular brain.

[20] Ritchie L, Tate R, Chamberlain LH, Robertson G, Zagnoni M, Sposito T, Wray S, Wright JA, Bryant CE, Gay NJ, Bushell TJ (2018). Toll-like receptor 3 activation impairs excitability and synaptic activity via TRIF signalling in immature rat and human neurons. Neuropharmacology.

[21] Krishnan V, Nestler EJ (2011). Animal models of depression: molecular perspectives. Curr Top Behav Neurosci.

[22] Kessing CF, Tyor WR (2015). Interferon-alpha induces neurotoxicity through activation of the type I receptor and the GluN2A subunit of the NMDA receptor. J Interf Cytokine Res.

[23] Zheng LS, Hitoshi S, Kaneko N, Takao K, Miyakawa T, Tanaka Y, Xia H, Kalinke U, Kudo K, Kanba S, Ikenaka K, Sawamoto K (2014). Mechanisms for interferon-alpha-induced depression and neural stem cell dysfunction. Stem Cell Reports.

[24] Giovanoli S, Weber-Stadlbauer U, Schedlowski M, Meyer U, Engler H (2016). Prenatal immune activation causes hippocampal synaptic deficits in the absence of overt microglia anomalies. Brain Behav Immun.

[25] Patrich E, Piontkewitz Y, Peretz A, Weiner I, Attali B (2016). Maternal immune activation produces neonatal excitability defects in offspring hippocampal neurons from pregnant rats treated with poly I:C. Sci Rep.

[26] Hepgul N, Cattaneo A, Agarwal K, Baraldi S, Borsini A, Bufalino C, Forton DM, Mondelli V, Nikkheslat N, Lopizzo N, Riva MA, Russell A, Hotopf M, Pariante CM (2016). Transcriptomics in interferon-alpha-treated patients identifies inflammation-, neuroplasticity- and oxidative stress-related signatures as predictors and correlates of depression. Neuropsychopharmacology.

[27] Zheng LS, Kaneko N, Sawamoto K (2015). Minocycline treatment ameliorates interferon-alpha- induced neurogenic defects and depression-like behaviors in mice. Front Cell Neurosci.

[28] Taylor MJ, Godlewska B, Near J, Christmas D, Potokar J, Collier J, Klenerman P, Barnes E, Cowen PJ (2014). Effect of interferon-alpha on cortical glutamate in patients with hepatitis C: a proton magnetic resonance spectroscopy study. Psychol Med.

[29] Gasull-Camos J, Tarres-Gatius M, Artigas F, Castane A (2017). Glial GLT-1 blockade in infralimbic cortex as a new strategy to evoke rapid antidepressant-like effects in rats. Transl Psychiatry.

[30] Ehret M, Sobieraj DM (2014). Prevention of interferon-alpha-associated depression with antidepressant medications in patients with hepatitis C virus: a systematic review and meta-analysis. Int J Clin Pract.

[31] Kirvell SL, Esiri M, Francis PT (2006). Down-regulation of vesicular glutamate transporters precedes cell loss and pathology in Alzheimer’s disease. J Neurochem.

[32] Hu S, Sheng WS, Ehrlich LC, Peterson PK, Chao CC (2000). Cytokine effects on glutamate uptake by human astrocytes. Neuroimmunomodulation.

[33] Costello DA, Lynch MA (2013). Toll-like receptor 3 activation modulates hippocampal network excitability, via glial production of interferon-beta. Hippocampus.

[34] Scumpia PO, Kelly KM, Reeves WH, Stevens BR (2005). Double-stranded RNA signals antiviral and inflammatory programs and dysfunctional glutamate transport in TLR3-expressing astrocytes. Glia.

[35] Fremeau RT, Kam K, Qureshi T, Johnson J, Copenhagen DR, Storm-Mathisen J, Chaudhry FA, Nicoll RA, Edwards RH (2004). Vesicular glutamate transporters 1 and 2 target to functionally distinct synaptic release sites. Science.

[36] Mendoza-Fernandez V, Andrew RD, Barajas-Lopez C (2000). Interferon-alpha inhibits long-term potentiation and unmasks a long-term depression in the rat hippocampus. Brain Res.

[37] Dedoni S, Olianas MC, Ingianni A, Onali P (2012). Type I interferons impair BDNF-induced cell signaling and neurotrophic activity in differentiated human SH-SY5Y neuroblastoma cells and mouse primary cortical neurons. J Neurochem.

[38] Gibney SM, McGuinness B, Prendergast C, Harkin A, Connor TJ (2013). Poly I:C-induced activation of the immune response is accompanied by depression and anxiety-like behaviours, kynurenine pathway activation and reduced BDNF expression. Brain Behav Immun.

[39] Kenis G, Prickaerts J, van Os J, Koek GH, Robaeys G, Steinbusch HW, Wichers M (2011). Depressive symptoms following interferon-alpha therapy: mediated by immune-induced reductions in brain-derived neurotrophic factor?. Int J Neuropsychopharmacol.

