# Supplementary material for: Compromised Hippocampal Neuroplasticity in the Interferon-α and Toll-like Receptor-3 Activation-Induced Mouse Depression Model
Source: Mol Neurobiol. 2020 Jun 5;57(7):3171–82. doi: 10.1007/s12035-020-01927-0 (PMC7320059; doi:10.1007/s12035-020-01927-0)
Supplement: Supplementary file 1 — IFN-α and poly(I:C) do not induce changes of dendritic spine length ex vivo. No significant differences were found of the length of dendritic spines in either (A) apical or (B) basal CA1 or (C) dentate gyrus neurons in Golgi-Cox stained sections from mice exposed to vehicle, IFN-α (250 IU/day), poly(I:C) (1 μg/day) or combined IFN-α and poly(I:C) (as before) delivery. Data are means ± S.D. No significant differences were noted between groups (≥10 randomly selected neurons per mouse evaluated in A, of which 2 apical and 2 basal dendrites each were examined in n = 5 mice/ group; tissue samples of n = 5 mice/ group separately evaluated in B as triplicates) (PPTX 82 kb) [file 12035_2020_1927_MOESM1_ESM.pptx]

## Slide 1
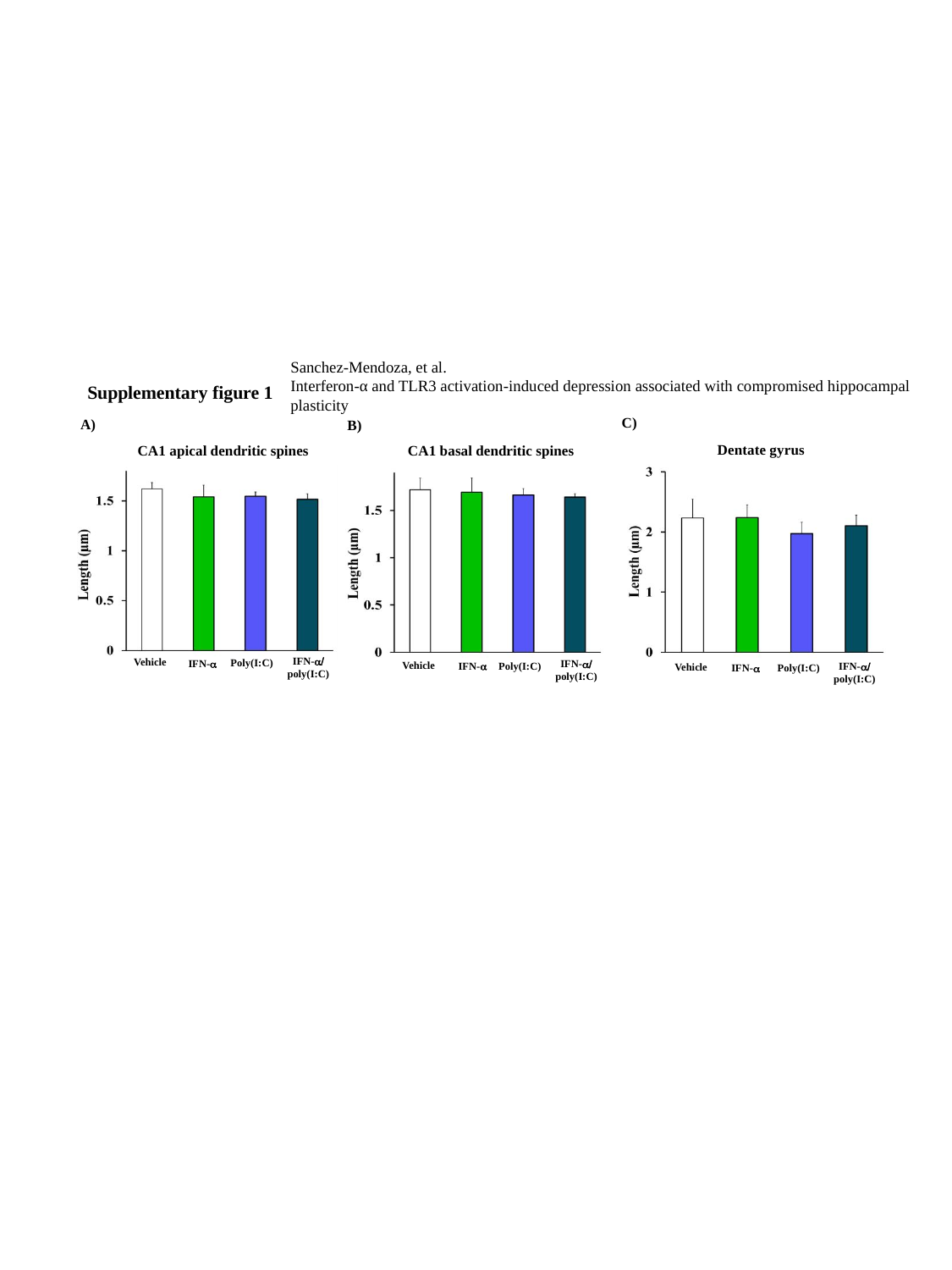

Sanchez-Mendoza, et al.
Interferon-α and TLR3 activation-induced depression associated with compromised hippocampal plasticity
Supplementary figure 1
Dentate gyrus
CA1 apical dendritic spines
CA1 basal dendritic spines
IFN-a/
poly(I:C)
Vehicle
Poly(I:C)
IFN-a
IFN-a/
poly(I:C)
Vehicle
IFN-a
Poly(I:C)
IFN-a/
poly(I:C)
Vehicle
IFN-a
Poly(I:C)
C)
A)
B)
