# Supplementary material for: Compromised Hippocampal Neuroplasticity in the Interferon-α and Toll-like Receptor-3 Activation-Induced Mouse Depression Model
Source: Mol Neurobiol. 2020 Jun 5;57(7):3171–82. doi: 10.1007/s12035-020-01927-0 (PMC7320059; doi:10.1007/s12035-020-01927-0)
Supplement: Supplementary file 2 — Poly(I:C) reduces BDNF expression ex vivo in the hippocampus. (A) Representative images of BDNF expressing cells in the hippocampus of mice exposed to vehicle, IFN-α (250 IU/day), poly(I:C) (1 μg/day) or combined IFN-α and poly(I:C) (as before) delivery. Squares show regions of interest in the CA1 and DG regions which were quantified. Poly(I:C), but not IFN-α or combined IFN-α and poly(I:C) delivery, significantly reduced the number of BDNF expressing cells in the CA1 region (B), but not the DG (C). Data are means ± SD, analyzed by one-way ANOVA, followed by Tukey post-hoc tests (n = 4–5 mice/group). **p = 0.01 compared to vehicle. (PPTX 804 kb) [file 12035_2020_1927_MOESM2_ESM.pptx]

## Slide 1
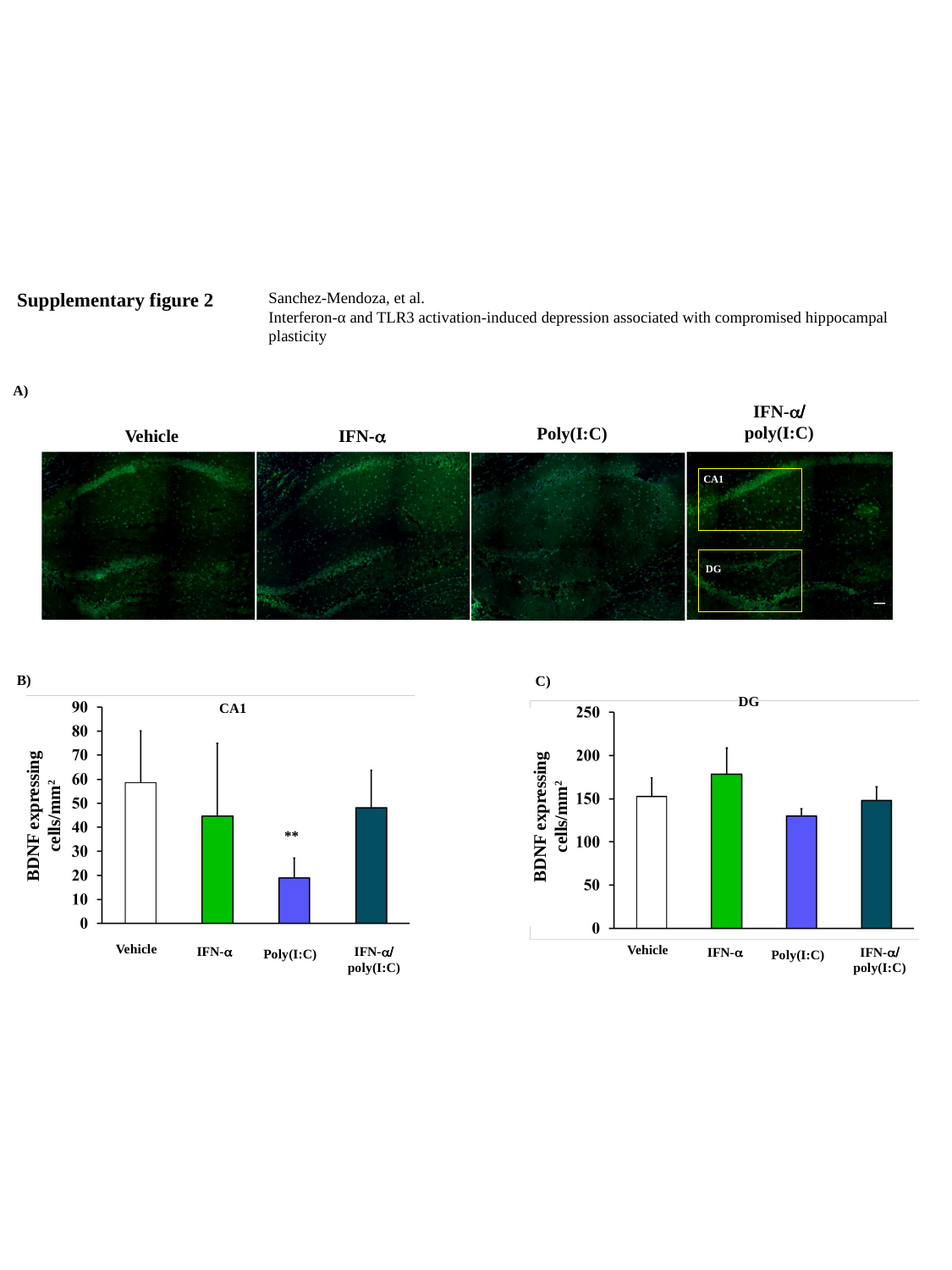

Supplementary figure 2
Sanchez-Mendoza, et al.
Interferon-α and TLR3 activation-induced depression associated with compromised hippocampal plasticity
A)
IFN-a/
poly(I:C)
Poly(I:C)
IFN-a
Vehicle
CA1
DG
B)
C)
DG
CA1
**
Vehicle
Vehicle
IFN-a
IFN-a/
poly(I:C)
IFN-a
IFN-a/
poly(I:C)
Poly(I:C)
Poly(I:C)
BDNF expressing cells/mm2
BDNF expressing cells/mm2
