# Supplementary material for: Compromised Hippocampal Neuroplasticity in the Interferon-α and Toll-like Receptor-3 Activation-Induced Mouse Depression Model
Source: Mol Neurobiol. 2020 Jun 5;57(7):3171–82. doi: 10.1007/s12035-020-01927-0 (PMC7320059; doi:10.1007/s12035-020-01927-0)
Supplement: Supplementary file 3 — IFN-α and poly(I:C) do not induce neurodegeneration or demyelination ex vivo. Neurofilament and myelin basic protein images were obtained from (A) the CA1 stratum lacunosum moleculare (slm) and (B) dentate gyrus polymorph layer (po) of mice exposed to vehicle, IFN-α (250 IU/day), poly(I:C) (1 μg/day) or combined IFN-α and poly(I:C) (as before) delivery. No significant differences of neurofilament or myelin basic protein density were found in (C, D) the CA1 region or (E, F) the dentate gyrus. Scale bars = 30 μm in A, 10 μm in B. (PPTX 741 kb) [file 12035_2020_1927_MOESM3_ESM.pptx]

## Slide 1
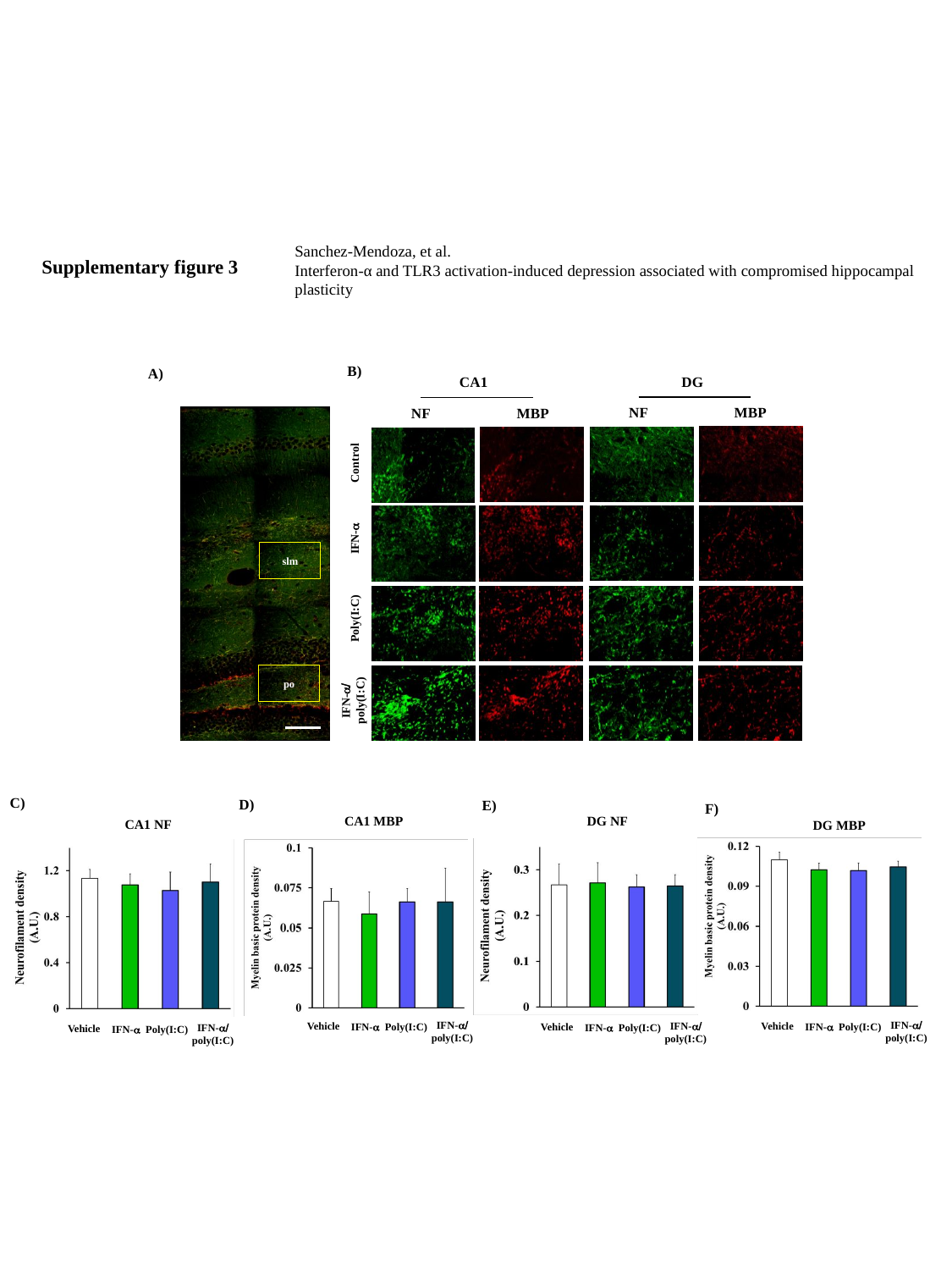

Sanchez-Mendoza, et al.
Interferon-α and TLR3 activation-induced depression associated with compromised hippocampal plasticity
Supplementary figure 3
B)
A)
DG
CA1
NF
MBP
NF
MBP
slm
po
Control
IFN-a
Poly(I:C)
IFN-a/
poly(I:C)
C)
D)
E)
F)
CA1 MBP
DG NF
CA1 NF
IFN-a/
poly(I:C)
Vehicle
IFN-a
Poly(I:C)
DG MBP
IFN-a/
poly(I:C)
IFN-a/
poly(I:C)
IFN-a/
poly(I:C)
Vehicle
Vehicle
IFN-a
Poly(I:C)
IFN-a
Poly(I:C)
Vehicle
IFN-a
Poly(I:C)
