# Supplementary material for: Compromised Hippocampal Neuroplasticity in the Interferon-α and Toll-like Receptor-3 Activation-Induced Mouse Depression Model
Source: Mol Neurobiol. 2020 Jun 5;57(7):3171–82. doi: 10.1007/s12035-020-01927-0 (PMC7320059; doi:10.1007/s12035-020-01927-0)
Supplement: Supplementary file 4 — IFN-α and poly(I:C) do not induce neuronal death ex vivo. No evidence of irreversibly injured, that is, TUNEL+ neurons was found in the CA1 region or in other areas of the hippocampus of mice exposed to vehicle, IFN-α (250 IU/day), poly(I:C) (1 μg/day) or combined IFN-α and poly(I:C) (as before) delivery. DAPI counterstainings are shown in blue. Scale bar = 20 μm. (PPTX 1088 kb) [file 12035_2020_1927_MOESM4_ESM.pptx]

## Slide 1
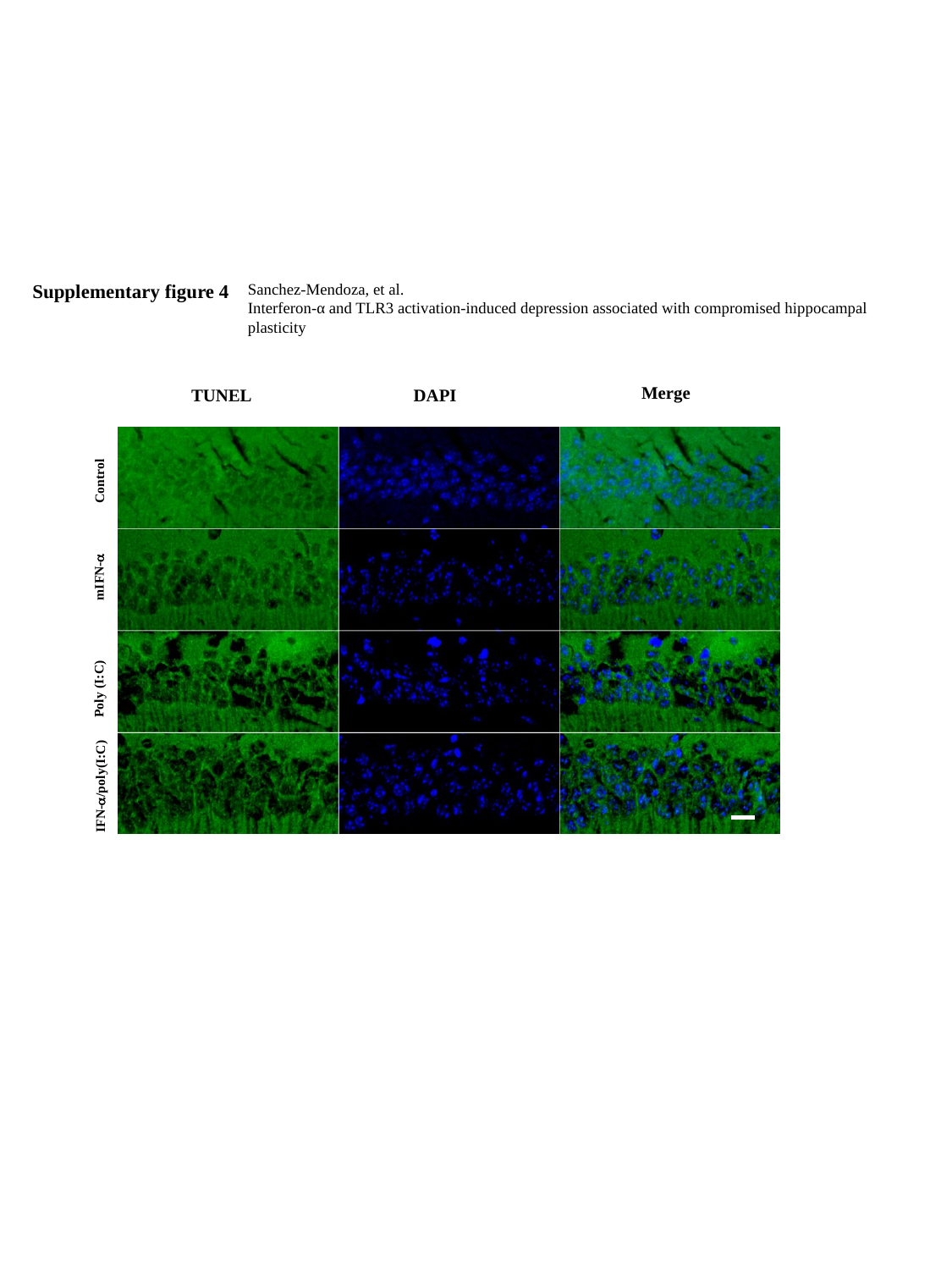

Sanchez-Mendoza, et al.
Interferon-α and TLR3 activation-induced depression associated with compromised hippocampal plasticity
Supplementary figure 4
Merge
TUNEL
DAPI
Control
mIFN-a
Poly (I:C)
IFN-a/poly(I:C)
