# Supplementary material for: Compromised Hippocampal Neuroplasticity in the Interferon-α and Toll-like Receptor-3 Activation-Induced Mouse Depression Model
Source: Mol Neurobiol. 2020 Jun 5;57(7):3171–82. doi: 10.1007/s12035-020-01927-0 (PMC7320059; doi:10.1007/s12035-020-01927-0)
Supplement: Supplementary file 5 — Representative Western blots for the presynaptic proteins VGLUT1 and VGLUT2 and the postsynaptic proteins PSD95, AMPAR1. Protein lysates were obtained from the hippocampus of mice exposed to vehicle, IFN-α (250 IU/day), poly(I:C) (1 μg/day) or combined IFN-α and poly(I:C) (as before) delivery. (PPTX 1213 kb) [file 12035_2020_1927_MOESM5_ESM.pptx]

## Slide 1
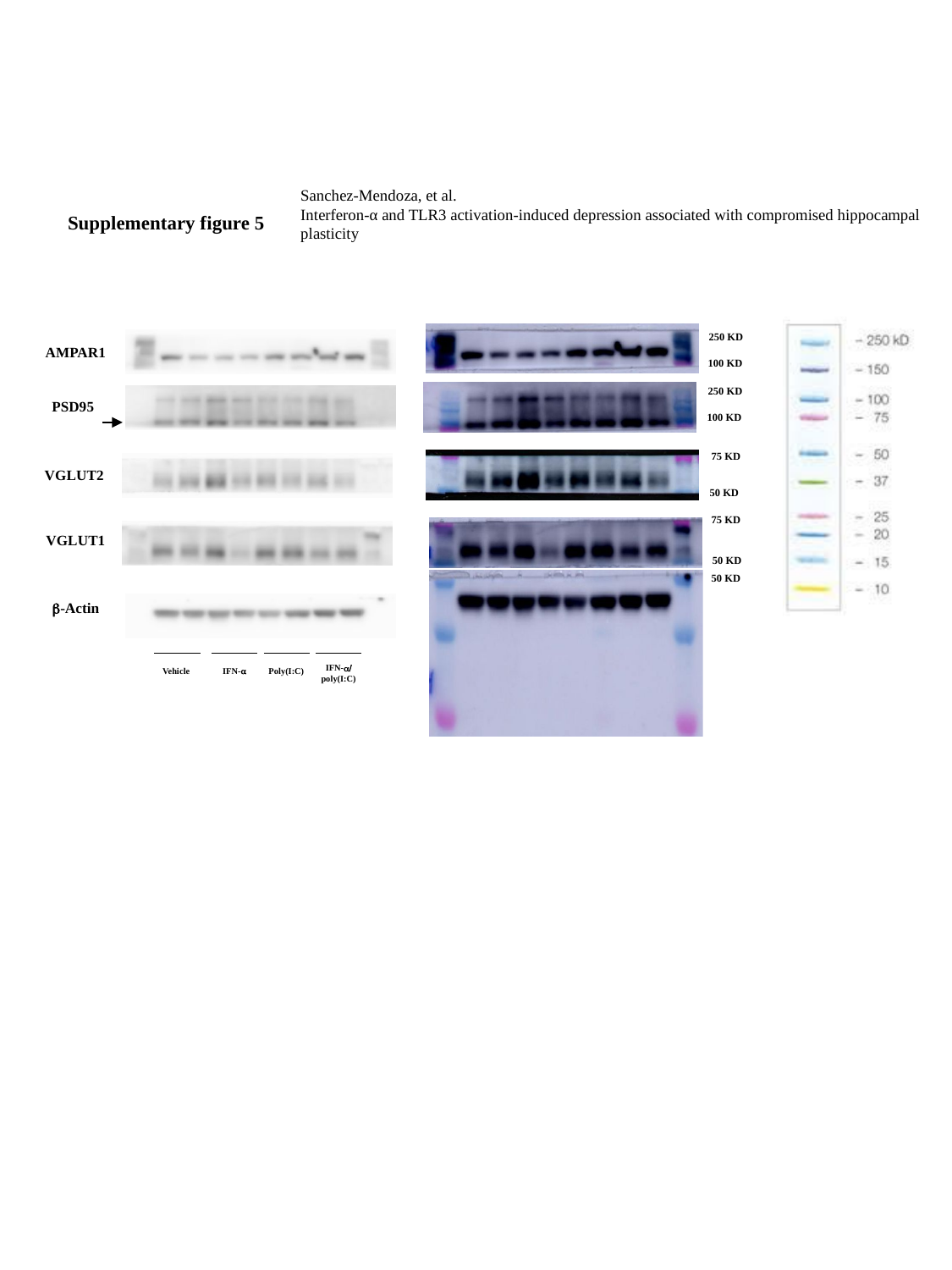

Sanchez-Mendoza, et al.
Interferon-α and TLR3 activation-induced depression associated with compromised hippocampal plasticity
Supplementary figure 5
250 KD
AMPAR1
100 KD
250 KD
PSD95
100 KD
75 KD
VGLUT2
50 KD
75 KD
VGLUT1
50 KD
50 KD
b-Actin
IFN-a/
poly(I:C)
Vehicle
IFN-a
Poly(I:C)
