# Supplementary material for: Compromised Hippocampal Neuroplasticity in the Interferon-α and Toll-like Receptor-3 Activation-Induced Mouse Depression Model
Source: Mol Neurobiol. 2020 Jun 5;57(7):3171–82. doi: 10.1007/s12035-020-01927-0 (PMC7320059; doi:10.1007/s12035-020-01927-0)
Supplement: Supplementary file 6 — IFN-α and poly(I:C) do not influence the presynaptic Ca++ sensor synaptotagmin-1 or the glutamate transporter EAAT2 ex vivo. (A) Synaptotagmin 1, which needs to be activated for synaptic vesicle fusion with the presynaptic membrane, or (B) EAAT2, which removes glutamate from or returns glutamate to the synaptic cleft, thus influencing glutamatergic transmission, were unchanged, as shown by Western blots of mice exposed to vehicle, IFN-α (250 IU/day), poly(I:C) (1 μg/day) or combined IFN-α and poly(I:C) (as before) delivery. Data are means ± S.D. No significant differences were observed between groups (n = 5 mice/ group evaluated as triplicates). (PPTX 196 kb) [file 12035_2020_1927_MOESM6_ESM.pptx]

## Slide 1
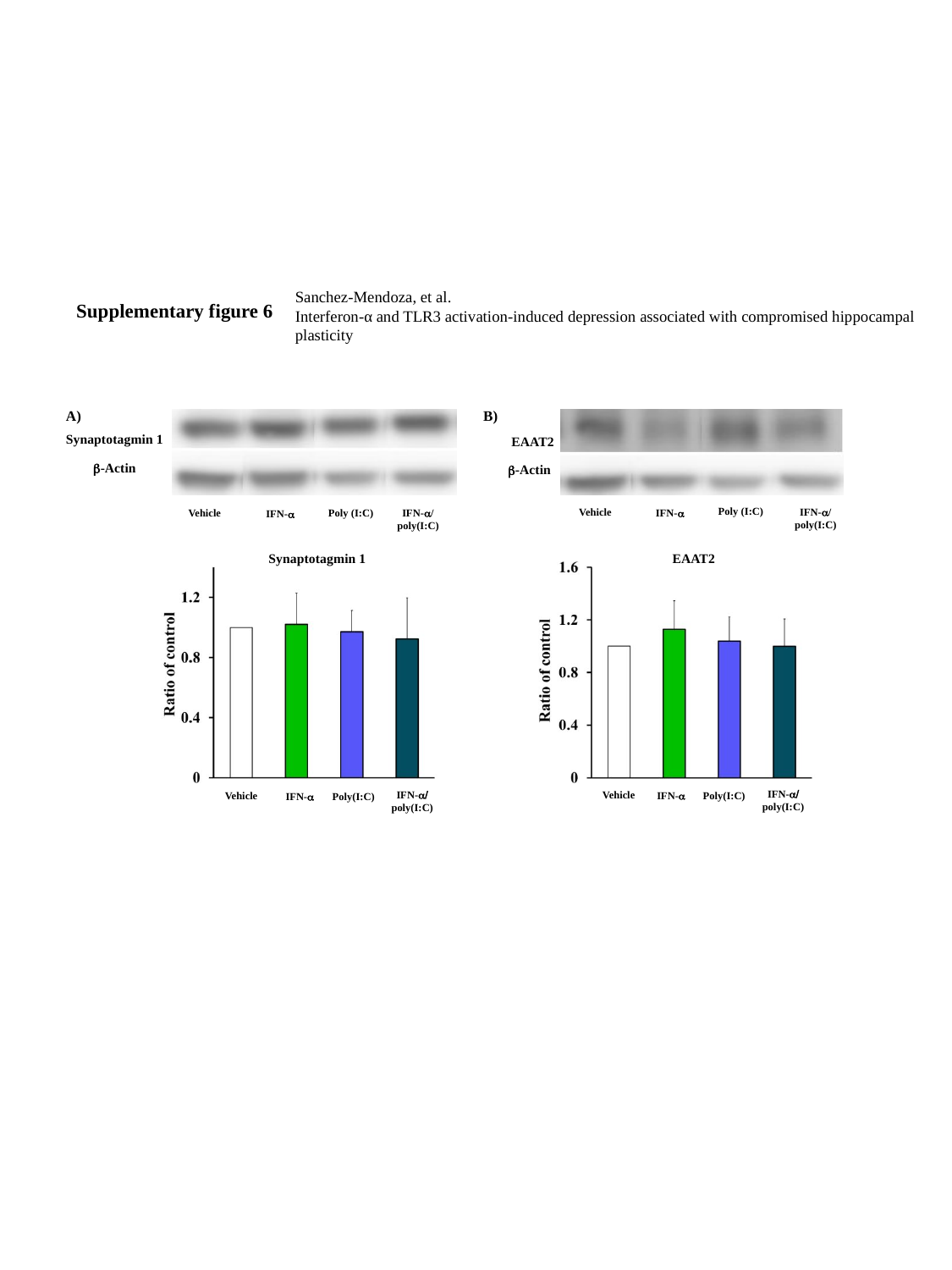

Sanchez-Mendoza, et al.
Interferon-α and TLR3 activation-induced depression associated with compromised hippocampal plasticity
Supplementary figure 6
A)
B)
Synaptotagmin 1
EAAT2
b-Actin
b-Actin
Poly (I:C)
IFN-a/
poly(I:C)
Vehicle
IFN-a
IFN-a/
poly(I:C)
Poly (I:C)
Vehicle
IFN-a
Synaptotagmin 1
EAAT2
IFN-a/
poly(I:C)
Vehicle
IFN-a
Poly(I:C)
IFN-a/
poly(I:C)
Vehicle
IFN-a
Poly(I:C)
