# Supplementary material for: Compromised Hippocampal Neuroplasticity in the Interferon-α and Toll-like Receptor-3 Activation-Induced Mouse Depression Model
Source: Mol Neurobiol. 2020 Jun 5;57(7):3171–82. doi: 10.1007/s12035-020-01927-0 (PMC7320059; doi:10.1007/s12035-020-01927-0)
Supplement: Supplementary file 7 — Phosphorylation of AMPAR1 does not change in vitro in response to IFN-α and poly(I:C) exposure. Phosphorylation level of AMPAR1 of primary hippocampal neurons depolarized for 1 h by 4-AP (2.5 mM) after exposure to vehicle, IFN-α (100 IU/mL), poly(I:C) (1 μg/mL) or IFN-α and poly(I:C) (as before). No significant changes of AMPAR1 phosphorylation were noted. Data are means ± S.D. (n = 3 experiments evaluated as triplicates). (PPTX 68 kb) [file 12035_2020_1927_MOESM7_ESM.pptx]

## Slide 1
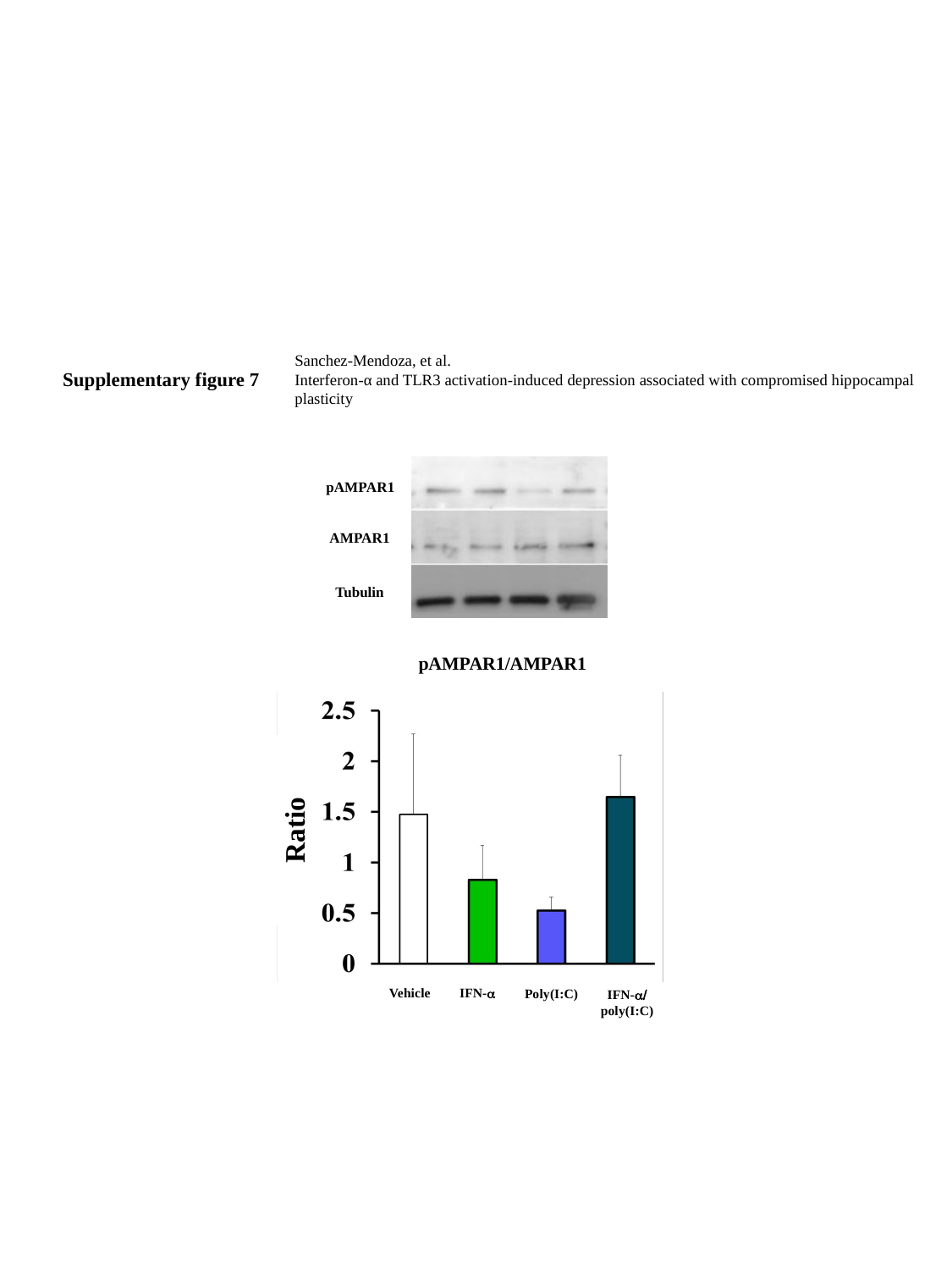

Sanchez-Mendoza, et al.
Interferon-α and TLR3 activation-induced depression associated with compromised hippocampal plasticity
Supplementary figure 7
pAMPAR1
AMPAR1
Tubulin
pAMPAR1/AMPAR1
Ratio
Vehicle
IFN-a
Poly(I:C)
IFN-a/
poly(I:C)
