# Supplementary material for: Compromised Hippocampal Neuroplasticity in the Interferon-α and Toll-like Receptor-3 Activation-Induced Mouse Depression Model
Source: Mol Neurobiol. 2020 Jun 5;57(7):3171–82. doi: 10.1007/s12035-020-01927-0 (PMC7320059; doi:10.1007/s12035-020-01927-0)
Supplement: Supplementary file 8 — Summary of findings. In response to IFN-α and poly(I:C) exposure, synaptic plasticity is compromised in CA1 apical dendrites. At the molecular level, reduced TrkB phosphorylation is accompanied by the decreased synthesis of the presynaptic protein VGLUT1 and postsynaptic protein PSD95. The altered synaptic plasticity is thought to contribute to IFN-α/ poly(I:C) associated depression. (PPTX 41 kb) [file 12035_2020_1927_MOESM8_ESM.pptx]

## Slide 1
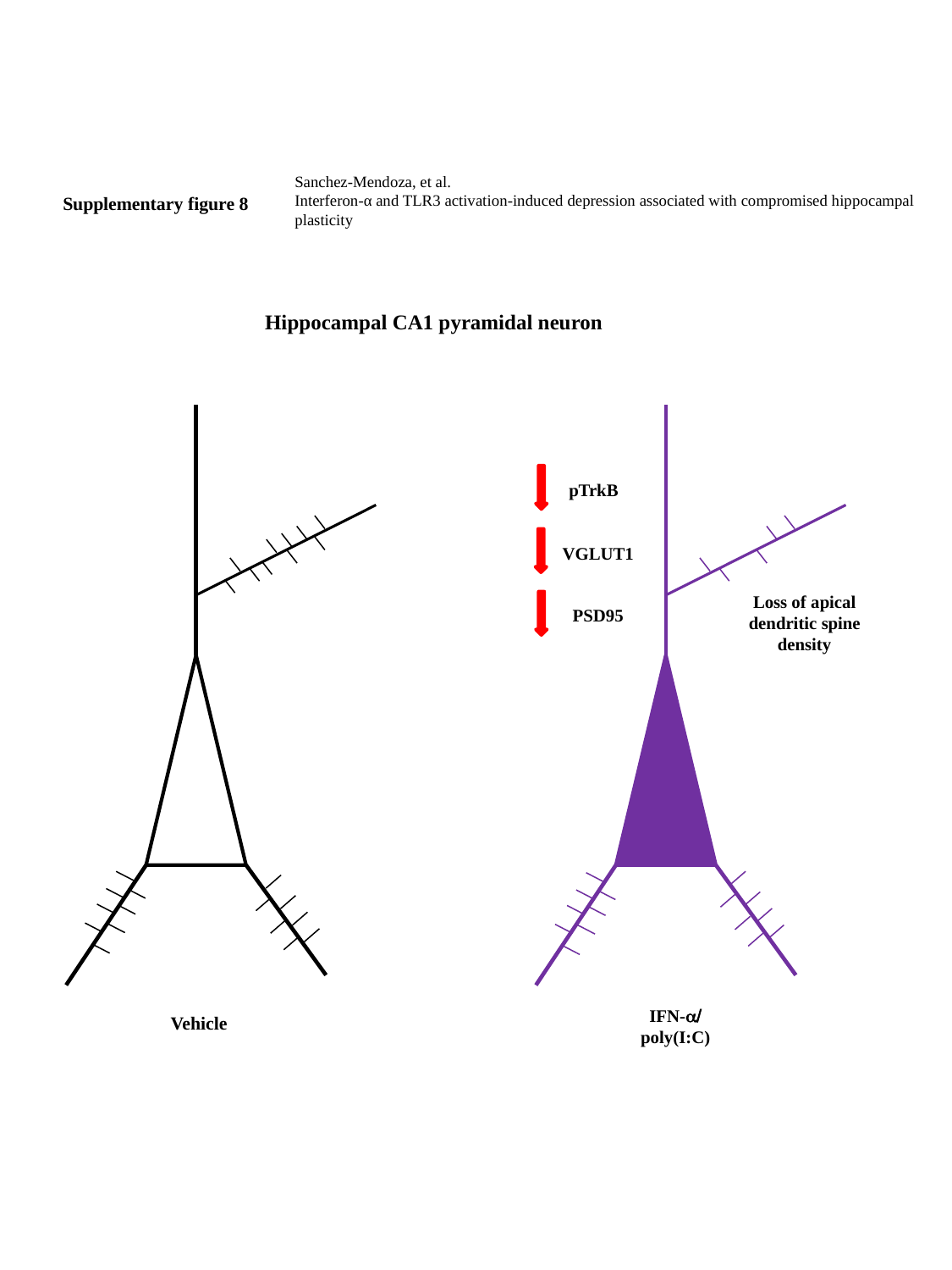

Sanchez-Mendoza, et al.
Interferon-α and TLR3 activation-induced depression associated with compromised hippocampal plasticity
Supplementary figure 8
Hippocampal CA1 pyramidal neuron
pTrkB
VGLUT1
Loss of apical dendritic spine density
PSD95
IFN-a/
poly(I:C)
Vehicle
